# Supplementary figures and images for: Identifying Key Genes as Progression Indicators of Prostate Cancer with Castration Resistance Based on Dynamic Network Biomarker Algorithm and Weighted Gene Correlation Network Analysis
Source: Biomedicines. 2024 Sep 23;12(9):2157. doi: 10.3390/biomedicines12092157 (PMC11429123; doi:10.3390/biomedicines12092157)

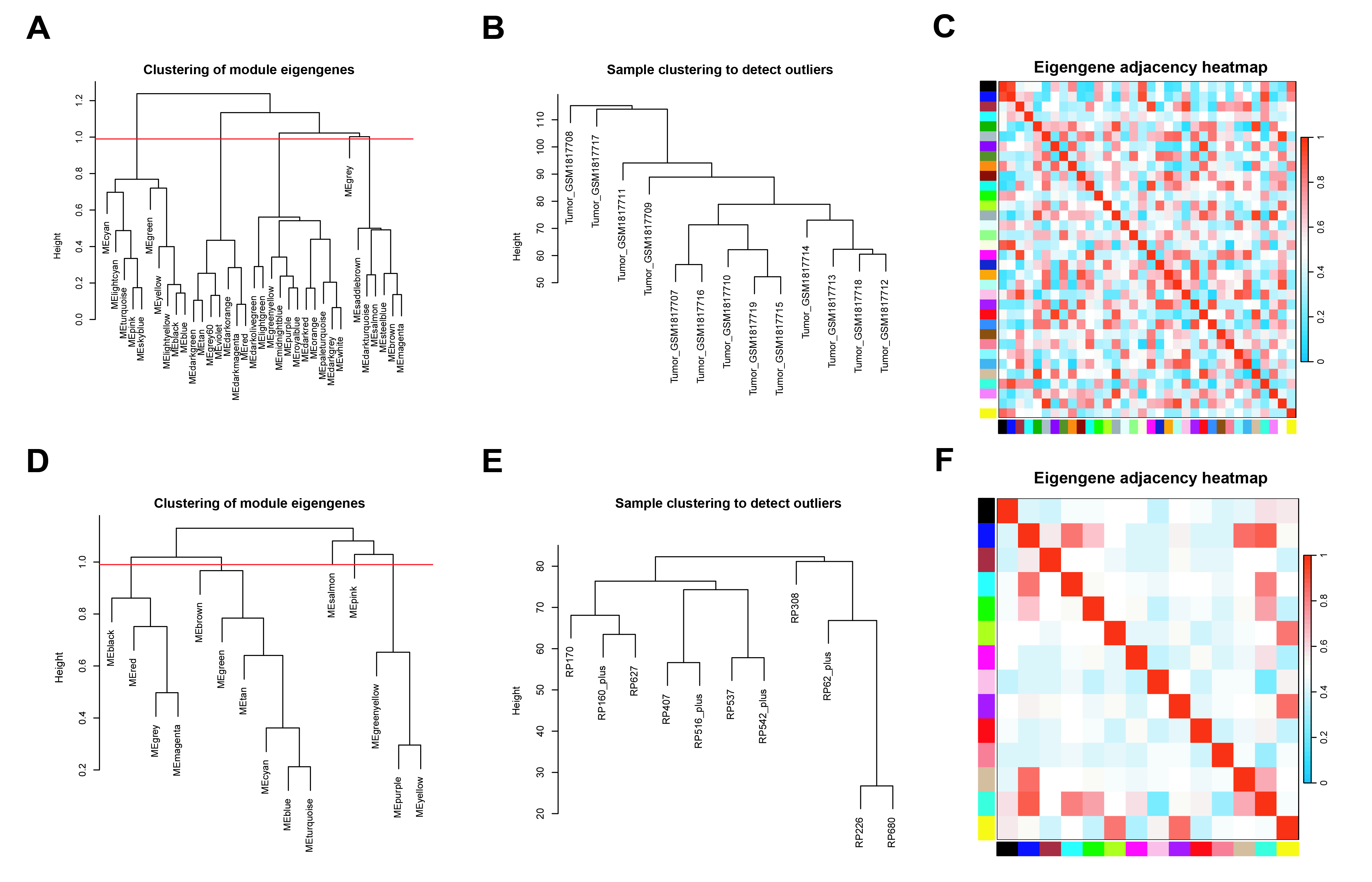

Supplement: Supplementary file 1 [file biomedicines-12-02157-s001.zip › Figure S3.tif]

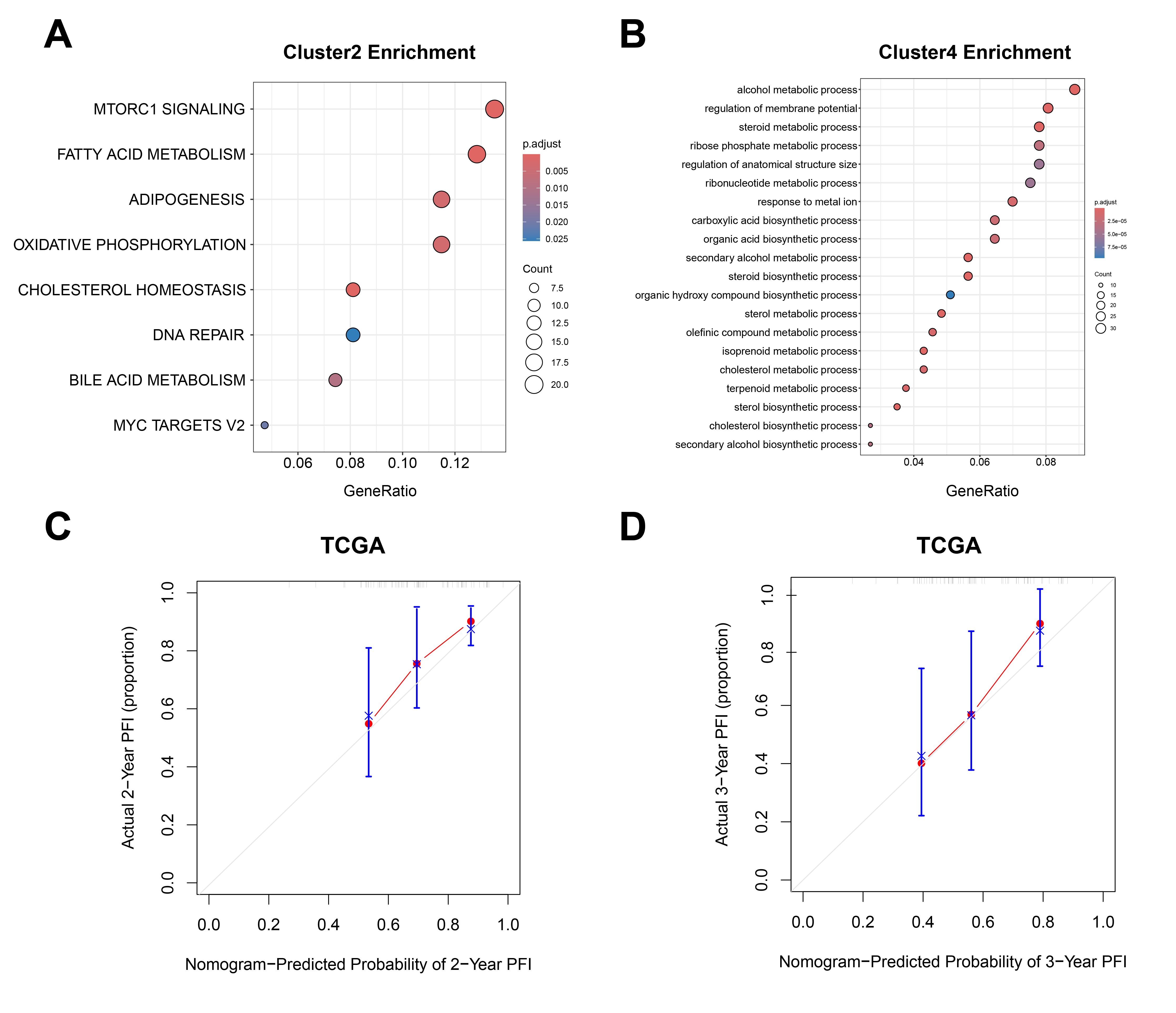

Supplement: Supplementary file 1 [file biomedicines-12-02157-s001.zip › Figure S4.tif]

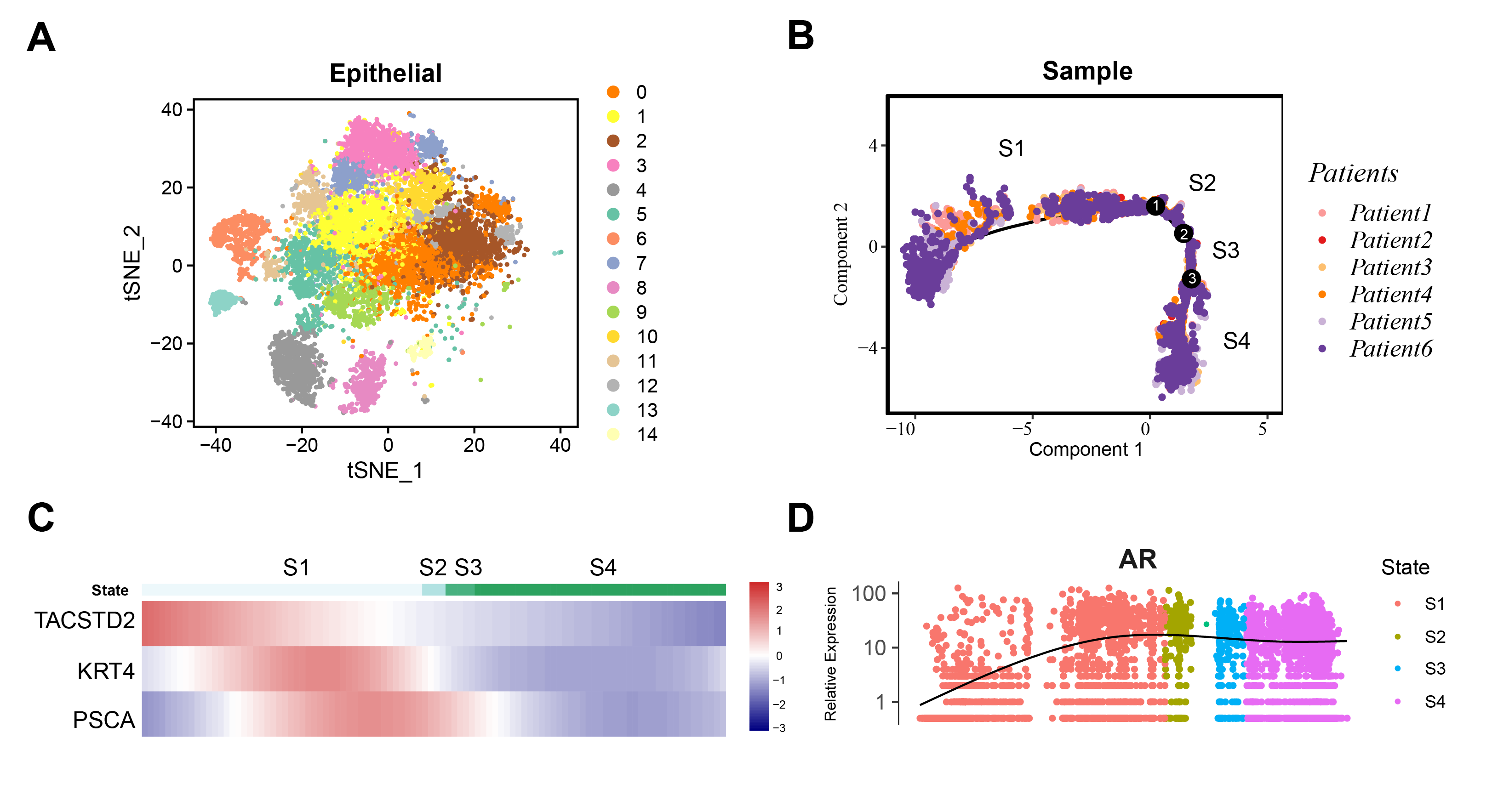

Supplement: Supplementary file 1 [file biomedicines-12-02157-s001.zip › Figure S1.tif]
